# Women with a History of Recurrent Pregnancy Loss Are a High-Risk Population for Adverse Obstetrical Outcome: A Retrospective Cohort Study

**DOI:** 10.3390/jcm10020179

**Published:** 2021-01-06

**Authors:** Emma Rasmark Roepke, Ole Bjarne Christiansen, Karin Källén, Stefan R. Hansson

**Affiliations:** 1 Institute of Clinical Sciences Lund, Lund University, 22100 Lund, Sweden; karin.kallen@med.lu.se (K.K.); stefan.hansson@med.lu.se (S.R.H.); 2 Department of Obstetrics and Gynaecology, Skåne University Hospital, Jan Waldenströmsgatan 47, 20502 Malmö, Sweden; 3 Centre for Recurrent Pregnancy Loss of Western Denmark, Department of Obstetrics and Gynaecology, Aalborg University Hospital, Clinical Institute at Aalborg University, 9220 Aalborg, Denmark; olbc@rn.dk

**Keywords:** recurrent pregnancy loss, pre-eclampsia, intrauterine growth restriction, preterm birth, stillbirth, placental abruption

## Abstract

Recurrent pregnancy loss (RPL), defined as three or more consecutive miscarriages, is hypothesized to share some of the same pathogenic factors as placenta-associated disorders. It has been hypothesized that a defect implantation causes pregnancy loss, while a partially impaired implantation may lead to late pregnancy complications. The aim of this retrospective register-based cohort study was to study the association between RPL and such disorders including pre-eclampsia, stillbirth, small for gestational age (SGA) birth, preterm birth and placental abruption. Women registered with childbirth(s) in the Swedish Medical Birth Register (MFR) were included in the cohort. Pregnancies of women diagnosed with RPL (exposed) in the National Patient Register (NPR), were compared with pregnancies of women without RPL (unexposed/reference). Obstetrical outcomes, in the first pregnancy subsequent to the diagnosis of RPL (*n* = 4971), were compared with outcomes in reference-pregnancies (*n* = 57,410). Associations between RPL and placental dysfunctional disorders were estimated by odds ratios (AORs) adjusting for confounders, with logistic regression. RPL women had an increased risk for pre-eclampsia (AOR 1.45; 95% CI; 1.24–1.69), stillbirth <37 gestational weeks (GWs) (AOR 1.92; 95% CI; 1.22–3.02), SGA birth (AOR 1.97; 95% CI; 1.42–2.74), preterm birth (AOR 1.46; 95% CI; 1.20–1.77), and placental abruption <37 GWs (AOR 2.47; 95% CI; 1.62–3.76) compared with pregnancies by women without RPL. Women with RPL had an increased risk of pregnancy complications associated with placental dysfunction. This risk population is, therefore, in need of improved antenatal surveillance.

## 1. Introduction

Recurrent pregnancy loss (RPL) is defined as two [1,2] or, traditionally more strictly, three or more consecutive miscarriages occurring before the fetus reaches viability [3]. The later definition of three or more miscarriages is used in this study since this definition is used in Sweden where the study is conducted. Other societies, such as the German, Austrian and Swiss Society for Obstetrics and Gynecology as well as the British Royal College for obstetrics and gynecology, support this definition [3,4].

RPL is distressing for the affected couples. About 0.5–2.3% [5,6,7,8,9,10] of women trying to conceive suffer RPL. The most common risk factors underlying RPL are: parental chromosomal aberrations, congenital and acquired uterine abnormalities, endocrine abnormalities, hereditary thrombophilia and autoimmune factors such as the antiphospholipid syndrome [11]. However, in about half of the cases, investigations for RPL do not reveal any known etiology [12,13,14]. Insufficient placentation on an immunological basis has been suggested as an additional explanation in this heterogeneous group [11,15].

Implantation and placentation are complex processes that depend on a two-way communication between the embryo and the mother [16,17], regulated by reciprocal signaling between decidua, immune cells, and the fetal trophoblast cells [18]. Vascular remodeling of the spiral arteries by the extravillous trophoblast cells, plays a key role in successful implantation and is crucial to secure a good pregnancy outcome [19,20]. It has been hypothesized that a defect implantation causes pregnancy loss, while a partially impaired implantation may lead to late pregnancy complications [20] such as pre-eclampsia, stillbirth, intrauterine growth restriction (IUGR), preterm birth and placental abruption [19,21,22,23]. As miscarriage and placental dysfunctional disorders seem to share some of the same pathophysiology, a history of RPL might be associated with an increased risk of placenta-associated disorders.

Several studies supporting the abovementioned theory report a significantly higher rate of pre-eclampsia [24,25,26], stillbirth [21,24,26,27], small for gestational age (SGA) infants [14,21,24,26,28], preterm delivery [14,21,24,26,27,28,29] and placental abruption [24,25,26] in women with three or more miscarriages.

Primary RPL is defined as no live birth before the recurrent miscarriages, and secondary RPL as at least one live birth before recurrent miscarriages. As a hypothesis, primary and secondary RPL might differ in etiology and, therefore, risk of obstetrical complication might differ [28]. This was recently emphasized in a large register study [30], finding women with primary RPL to have a significantly increased risk of long-term diseases following RPL, which was not seen in women with secondary RPL.

It is of great importance to know whether women with a history of RPL are at higher risk for obstetrical complications and, therefore, in need of closer surveillance during pregnancy.

In this large population-based register study, we aimed to investigate whether RPL is associated with a higher than normal obstetrical risk. In addition, we also aimed to compare pregnancy outcomes for primary and secondary RPL.

## 2. Material and Methods

This retrospective register-based cohort study used data from the Swedish Medical Birth Register (MFR) and the Swedish National Patient Register (NPR) provided by the National Board of Health and Welfare (NBHW). These registers can be linked at an individual level through a unique personal identity number given to each Swedish resident [31].

The MFR contains data on more than 97% of all births in Sweden since 1973 [32], including demographic data, information on reproductive history and complications during pregnancy, delivery, and the neonatal period. A midwife records information such as maternal weight, height, smoking habits, and medical and obstetric history from antenatal visits, which is then reported to the MFR. After delivery, the responsible physician or midwife records information about the women’s diseases and complications during pregnancy and delivery, according to the International Classification of Diseases (ICD), which is then forwarded to the MFR.

The NPR includes mandatory reported diagnosis codes, according to the ICD, from each of the 21 county councils in Sweden. Since 1987, the NPR has included all inpatient care in Sweden, and since 2001, the register has also covered outpatient visits, including both private and public caregivers. The underreporting of inpatient data has been estimated to be less than 1%. The rate of underreporting for outpatients has not been stated [33].

### 2.1. Study Population

The cohort included one group of women with childbirth and history of RPL (exposed) and another reference group of women with childbirth but without the diagnoses of RPL (unexposed). Women registered with ICD-10 codes of RPL, N96.9 or O26.2 in the NPR during the period 2003–2012, and their childbirths registered in MFR, were included. A reference group, without a history of RPL who had given birth, registered in the MFR during the above period, was randomly selected by the NBHW. Only singleton pregnancies were included. In this study, we focused only on the first subsequent childbirth, following the diagnosis of RPL. To be able to investigate RPL association to obstetric complications in a subsequent pregnancy, childbirths before the miscarriages were excluded. All births after the diagnoses of RPL could be of interest, although in this study, we focused only on the first sequent childbirth. These pregnancies with variable outcomes were compared with the pregnancies in the reference group. Women with primary and secondary RPL were identified. As there are different risks of obstetrical complications for nulliparous and multipara, women with primary RPL were compared with a nulliparous reference group and women with secondary RPL were compared with a multipara reference group to explore differences in obstetrical outcomes in primary vs. secondary RPL.

The quality of the diagnosis of RPL in the NPR has been evaluated with a positive predictive value (PPV) of 85% (95% CI; 78–89%). A negative predictive value could not be estimated due to the study design [34].

### 2.2. Outcome Variables

The main outcome measures were late pregnancy complications: pre-eclampsia, stillbirth, SGA birth, spontaneous preterm birth and placental abruption.

Pre-eclampsia was defined through ICD-10 codes, O14-O15, as a rise in blood pressure (BP) (>140/90 mm Hg) combined with proteinuria (>0.3 g/24 h or +1 or more on dipstick) on at least two occasions after the 20th week of gestation. A classification of severe pre-eclampsia was given if one of the following occurred: systolic BP ≥ 160 mm Hg, diastolic BP ≥ 110 mm Hg, oliguria (<500 mL/24 h), cerebral or visual symptoms, pulmonary edema, severe epigastric pain, thrombocytopenia (<100 × 10^9^/L), or increased liver enzymes (twice upper normal level) [35].

The quality of the diagnosis of pre-eclampsia in the MFR has been validated with a PPV of 93% [36].

Stillbirth was defined as fetal death after 22 completed gestational weeks (GWs) until delivery; though before July 2008, the limit was after 28 GWs until delivery. The midwife or physician assisting at the delivery registered stillbirth in a check box in the MFR, which was used throughout the study. Fetal death during delivery was excluded from the analysis.

The term SGA was used and was considered to equate to IUGR and defined as birth weight less than two standard deviations below the mean for the GWs according to growth and development record charts [37]. Information on SGA or appropriate for gestational age was missing in 313 (0.5%) births, which were assumed to be AGA. Both live born and stillborn children were included in the analyses.

Preterm birth was defined as a birth before 37 completed GWs. The gestational age was determined by routine ultrasound scans at around 18–21 GWs (97% of pregnant women) but if this was not available, the last menstrual period was used for determining the gestational age [38]. Very preterm birth was defined as a birth at 32 GWs or before, and moderately preterm births from 32 to 36 GWs. Preterm birth was further subdivided into those who had a spontaneous or iatrogenic start of delivery. At delivery, the responsible midwife records the start of labor, which is labeled as spontaneous labor, induced or Caesarean section, which makes it possible to identify women with spontaneous start. We did not have information about preterm premature rupture of membranes. Those with missing details on delivery start (4.7%) were assumed to have had a spontaneous start. Those with missing details of gestational age at birth were assumed as term birth.

Placental abruption was defined through the ICD-10 code O45 as the separation of the placenta from the maternal uterine attachment. Clinically, signs are uterine bleeding and uterine muscle hypertonia with consequences as fetal distress or fetal death.

Information on the following maternal characteristics was extracted from the MFR: maternal age, body mass index (BMI), nationality, smoking habits and assisted reproduction. Nationality was categorized into women born in Nordic countries (Sweden, Norway, Finland, Denmark and Iceland) and non-Nordic countries (all others). Smokers were defined as women who were current smokers at the time of attendance at their first antenatal visit. Assisted reproduction included in vitro fertilization (IVF) and intra-cytoplasmic sperm injection (ICSI).

Data were extracted for medical disorders diagnosed with the ICD-10 codes or in check boxes in the MFR. The medical disorders included chronic hypertension (ICD-10 code O10), pre-gestational diabetes mellitus (type 1 ICD-10 code O24.0 and type 2 ICD-10 code O24.1), asthma (ICD-10 code J45), cardiac disease (ICD-10 codes I50–51), renal disease (ICD-10 codes I12, N15), systemic lupus erythematosus (SLE) (ICD-10 code M32), antiphospholipid syndrome (APLS) (ICD-10 code D68.6), thrombophilia (ICD-10 codes D68), inflammatory bowel disease (IBD) (ICD-10 codes K50–51) and hypothyroidism (ICD-10 code E03).

### 2.3. Statistics

The association between RPL and the risk of pre-eclampsia, stillbirth, SGA birth, spontaneous preterm birth and placental abruption were estimated in the first pregnancy after the diagnosis of RPL. A comparison group without a history of RPL was used as a reference.

Basic descriptive statistics were used to describe maternal characteristics, and a comparison between the study and the reference group plus primary and secondary RPL group was made using the χ^2^-test. The risks of adverse obstetrical outcome according to history of RPL were approximated by odds ratios (ORs) with 95% confidence intervals (CIs). Since there were low initial risk of the obstetrical outcomes, ORs could be used equivalent to relative risk. Subgroup analyses on women with primary RPL and secondary RPL explored differences in the risk of obstetrical outcomes. Adjustment for confounding factors was obtained by estimating the adjusted OR (AOR) in a stepwise unconditional multiple logistic regression analysis. The following variables were included, when relevant, in our regression model: maternal age, early pregnancy, BMI, smoking habits, the mother’s nationality, assisted fertilization, chronic hypertension, and pre-gestational diabetes. These factors were chosen because of their known or potential association with RPL and adverse obstetrical outcomes. The regression model was adjusted to relevant confounder variables for each different outcome. Factors with a univariable association with the outcome, *p* < 0.2, were first selected for a multiple model. Then, a restricted model including only variables with *p* < 0.2 was assessed.

Missing values were handled with imputed mean for BMI. This is eligible since the frequency of missing data is low (about 10%), thus multiple imputations will not play a significant difference. Women with no data on smoking habits were dealt with as a separate variable in the model. Managing missing data for SGA and preterm birth have been explained previously.

A value of *p* < 0.05 was considered significant. All analyses were performed using the SPSS version 24 (Armonk, NY, USA: IMB Corp.)

### 2.4. Ethics

The study was approved by one of the regional ethical review boards in Lund, Sweden, with reference number 2014/1. The ethical board waived the need for patient consent.

## 3. Results

This study included 62,381 pregnancies for analyses. There were a total of *n* = 4971 first subsequent pregnancies by women with RPL (exposed). The NBHW enabled to deliver a random selection of about 5% of all childbirths in Sweden in the study period, which resulted in the conducted unexposed group (*n* = 57,410 pregnancies) by women without RPL. Pregnancies by women with RPL (*n* = 4971) were compared with pregnancies by women without RPL (*n* = 57,410).

Of the 4971 women with a history of RPL and their first subsequent pregnancy in MFR, 2334 (47%) and 2637 (53%) had primary and secondary RPL, respectively.

### 3.1. Descriptive Statistics

Compared with women without a history of RPL, women with RPL were older, had a higher BMI and lower parity. They were more likely to have gone through assisted reproduction, to have chronic hypertension, hypothyroidism, asthma, APLS and pre-gestational diabetes. Women with RPL were less likely to be smokers and less were born in a Nordic country (Table 1.)

Compared to women with secondary RPL, women with primary RPL were younger and were less overweight. Women with primary RPL were more likely to have hypothyroidism and had more often undergone assisted reproduction than women with secondary RPL (Table 1).

### 3.2. Obstetrical Outcome

#### 3.2.1. Pre-Eclampsia

Compared with the reference group, women with a history of RPL had a higher risk of pre-eclampsia (4.3% vs. 2.2%; AOR 1.45; 95% CI; 1.24–1.69). The same results were seen after dividing pre-eclampsia into mild/moderate and severe. When occurrence of pre-eclampsia was divided into those with preterm and term birth, the association between a history of RPL and pre-eclampsia was much stronger in RPL patients with preterm birth, showing a twofold increased risk compared to women without RPL (AOR 2.26; 95% CI; 1.70–3.01) (Table 2b).

#### 3.2.2. Stillbirth

The risk of preterm stillbirth, but not of stillbirth at term, was significantly higher in women with RPL compared to references. The rates of a preterm stillbirth were 0.5% vs. 0.2% in RPL women vs. reference women, respectively (AOR 1.92; 95% CI; 1.22–3.02) (Table 2b).

#### 3.2.3. Small for Gestational Age

Compared to the reference group, women with RPL had a higher risk of having a SGA infant when it was born preterm but not when the gestational age was over 37 GWs: 1.5% vs. 0.6% and 1.9% vs. 1.9%, respectively. The AOR for a preterm SGA infant was 2.00 (95% CI; 1.54–2.60) (Table 2b).

#### 3.2.4. Preterm Birth

Women with a history of RPL had a significantly higher risk for preterm birth than women without RPL. The difference in risk was highest for very preterm birth (<32 GWs) with AOR 3.86 (95% CI; 3.16–4.72) (Table 2a).

#### 3.2.5. Placental Abruption

There was a higher rate of placental abruption in women with a history of RPL, 1.0% vs. 0.4% AOR 2.45 (95% CI; 1.78–3.38). There was no change in the above risk in patients with preterm vs. term birth (Table 2a).

#### 3.2.6. Primary vs. Secondary RPL

The obstetrical outcomes for women with primary RPL were compared to nulliparous in the reference group, and women with secondary RPL were compared with multiparous in the reference group (Table 3). The risk of pre-eclampsia, preterm birth and placental abruption were higher in both primary and secondary RPL compared with nulliparous and multiparous references. The risk of SGA was significantly higher in the secondary RPL group compared with the reference group (AOR 1.44 (95% CI; 1.11–1.88)) but not for the primary RPL group (AOR 1.15 (95% CI; 0.92–1.43)).

## 4. Discussion

### 4.1. Main Findings

Our study found RPL to be associated with an increased risk of placenta-associated disorders. Compared to a reference group without RPL, the women with a history of RPL had an increased risk of pre-eclampsia, preterm stillbirth (<37 GWs), SGA birth, preterm birth and placental abruption in the first pregnancy subsequent to the diagnosis of RPL. This association was stronger when the birth was preterm compared to term. Our results support the hypothesis that RPL and placental-associated disorders have a partially shared pathogenesis during implantation and placentation.

### 4.2. Strengths and Limitations

The major strength of this study is the large national register-based population with a reference group from the same register. The large number of cases allowed analysis of less common events, such as extreme preterm birth, stillbirth and placental abruption. It also made it possible to subdivide the obstetrical outcomes into preterm and term disorders.

Some studies have used self-reported miscarriages to identify women with RPL [24,39] which can entail a risk of bias. To minimize recall bias, this study used ICD-10 codes of RPL, registered in the NPR by physicians, which were validated with a high PPV (85%) [34]. Another strength was the ability to adjust for important confounders such as maternal age, smoking, BMI, conception by assisted reproduction and pre-gestational disorders; this ability is often lacking in other studies [25,40]. However, information about other potential confounders such as maternal socio-economic class and fetal sex were not available, and other residual confounders may still persist.

The main weaknesses in this study are its retrospective design and the potential missing register data. The RPL group is heterogeneous with several underlying causes and it would therefore be of further interest to analyze whether subgroups with different potential causes of RPL differ in terms of obstetrical outcomes, as well as to analyze if there is a difference in obstetrical outcome for women with known and unknown RPL, as some known causes, like APLS, can act as a confounder. APLS was possible to identify and another analysis was made to adjust for this but no difference in the odds ratio was seen. Other causes of RPL were, unfortunately, not possible to detect in this study due to lack of information about different RPL risk factors.

### 4.3. Interpretation

Placental dysfunctional disorders can have severe consequences in pregnancy, affecting both mother and infant. Therefore, it is of great importance to study associations between potential risk groups, such as women with a history of RPL, and placental dysfunctional disorders. A study by Gunnarsdottir et al. [24] investigated RPL and pre-eclampsia. They found that the strongest association for pre-eclampsia was in patients with preterm birth <37 GWs (AOR 1.62; 95% CI; 1.11–1.24) rather than in those with term births. Trogstad et al. [39] found an increased risk for pre-eclampsia in RPL but only if there was a history of assisted reproduction [39]. Two other studies [27,41] have failed to detect a higher risk for pre-eclampsia in RPL women, which might be explained by the small population (42 of women with RPL) [41] and the lack of a strict definition of consecutive miscarriages [27]. Our study showed an increased prevalence of pre-eclampsia in women with RPL (4.3%) compared to controls (2.2%). The association with RPL was strongest for preterm and severe pre-eclampsia (AOR 2.26; 95% CI; 1.70–3.01 and AOR 1.57; 95% CI; 1.20–2.07, respectively), which was in accordance with results published by Sheiner et al. [25]. These results support the use of increased antenatal surveillance in RPL and possible preventive treatment for pre-eclampsia with low-dose aspirin [42].

Furthermore, our study identified a higher risk of stillbirth, preterm delivery and SGA birth in RPL women compared with the reference group. This was equally in accordance with several other studies [13,21,22,25,27,40,43]. Increased risk for stillbirth and SGA infants was found in preterm birth (<37 GWs), although not at term (≥37 GWs) in our study, in line with the results of Gunnarsdottir et al. [24]. Additionally, we discovered the strongest association between RPL and extreme preterm delivery (<32 GWs), also reported by others [24,27]. These results support the view that antenatal care should screen women with RPL for IUGR in the third trimester. It is well established that aspirin is beneficial in reducing the risk of pre-eclampsia, IUGR, perinatal death and rates of spontaneous preterm birth in women with historical risk factors [44,45,46]. Future studies need to evaluate whether prophylactic treatment with aspirin can prevent or decrease the risk of these complications in women with a history of RPL.

Placental abruption is a less common event during pregnancy. We found evidence for a strong association, with an AOR > 2 for placental abruption comparing RPL with the controls. As several other studies have supported our findings of an association between placental abruption [24,25] and RPL, there is good evidence to manage women with RPL as an obstetrical risk population.

When comparing primary RPL and secondary RPL with reference nulliparous and multiparous, respectively, our results indicated that both primary and secondary RPL was associated with an increased risk of premature birth, pre-eclampsia and placental abruption. Association between primary RPL and preterm and SGA birth has been presented previously [28,40], although we could only find an significant association between secondary RPL and SGA birth. Further studies need to investigate whether obstetrical outcome differs between primary and secondary RPL, along with different etiologies of RPL.

## 5. Conclusions

We found a clear association between RPL and adverse late pregnancy outcomes, probably related to a defective placentation. There is evidence for managing women with RPL as a risk population for placenta-associated complications. This is presently not routinely done in Swedish antenatal care. However, detailed suggestions for a surveillance program are beyond the scope of this study. However, RPL women seem to be in need of increased antenatal surveillance to reduce the risk of pregnancy complications. Hopefully, enhanced antenatal care with better screening of the obstetrical history can help to decrease placental dysfunctional disorders. After clinical implementation of a surveillance program addressing risk factors, future studies are needed to evaluate the effect of increased antenatal care in women with RPL, including prophylactic treatment.

## Figures and Tables

**Table 1:** Characteristics of women with RPL (recurrent pregnancy loss) and women without RPL at each pregnancy identified in the Swedish Medical Birth Register and the Swedish National Patient Register.

Maternal Characteristics	RPL ^a^ *n* = 4971 (%)	No RPL ^a^ *n* = 57410 * (%)	*p*-Value ^b^	Primary ^a^ *n* = 2335 (%)	Secondary ^a^ *n* = 2636 (%)	*p*-Value ^c^
Age ^b,c^						
<25	303 (6.1)	11,621 (20.2)	<0.001	226 (9.7)	77 (2.9)	<0.001
25–29	907 (18.2)	15,541 (27.1)		532 (22.8)	375 (14.2)	
30–34	1637 (32.9)	17,073 (29.7)		815 (34.9)	822 (31.2)	
35–39	1499 (30.2)	10,493 (18.3)		541 (23.2)	958 (36.3)	
>40	625 (12.6)	2682 (4.7)		221 (9.5)	404 (15.3)	
Parity ^b^						
1	2335 (47.0)	24,510 (42.7)	<0.001	2335 (100)	0 (0.0)	-
2	1806 (36.3)	20,579 (35.8)		-	1806 (68.5)	
3	572 (11.5)	8025 (14.0)		-	572 (21.7)	
4+	258 (5.2)	4296 (7.5)		-	258 (9.8)	
BMI ^b,c^						
<18.5	64 (1.3)	1407 (2.5)	<0.001	37 (1.6)	27 (1.0)	<0.001
18.5–24.9	2450 (49.3)	29,964 (52.2)		1192 (51.0)	1258 (47.7)	
25.0–29.9	1314 (26.4)	12,187 (21.2)		557 (23.9)	757 (28.7)	
>30.0	695 (14.0)	5494 (9.6)		312 (13.4)	383 (14.5)	
Missing (or not recorded)	448 (9.0)	8358 (14.6)		237 (10.1)	211 (8.0)	
Smoking ^b^ First antenatal visit						
Yes	300 (6.0)	5805 (10.1)	<0.001	126 (5.4)	174 (6.6)	0.09
No	4417 (88.9)	48,412 (84.3)		2077 (89.0)	2340 (88.8)	
Missing (or not recorded)	254 (5.1)	3193 (5.6)		132 (5.7)	122 (4.6)	
Mother’s country of birth ^b^						
Nordic	3990 (80.3)	47067 (82)	<0.05	1897 (81.2)	2093 (79.4)	0.10
Non-nordic	981 (19.7)	10,343 (18.0)		438 (18.8)	543 (20.6)	
Assisted reproduction ^b^						
Yes	351 (7.1)	1327 (2.3)	<0.001	269 (11.5)	82 (3.1)	<0.001
No (or not recorded)	4620 (92.9)	56,083 (97.7)		2066 (88.5)	2554 (96.9)	
Chronic hypertension						
Yes	50 (1.0)	242 (0.4)	<0.001	18 (0.8)	32 (1.2)	0.12
No (or not recorded)	4921 (99.0)	57,168 (99.6)		2317 (99.2)	2604 (98.8)	
Pre-gestational diabetes ^b^						
Yes	43 (0.9)	314 (0.5)	<0.05	18 (0.8)	25 (0.9)	0.50
No (or not recorded)	4928 (99.1)	57,096 (99.5)		2317 (99.2)	2611 (99.1)	
Hypothyroidism ^b,c^						
Yes	211 (4.2)	528 (0.9)	<0.001	128 (5.5)	83 (3.1)	<0.001
No (or not recorded)	4760 (95.8)	56,882 (99.1)		2207 (94.5)	2553 (96.9)	
SLE						
Yes	9 (0.2)	55 (0.1)	0.072	3 (0.1)	6 (0.2)	0.41
No (or not recorded)	2962 (99.8)	57,355 (99.9)		2332 (99.9)	2630 (99.8)	
IBD						
Yes	41 (0.8)	353 (0.6)	0.073	18 (0.8)	23 (0.9)	0.69
No (or not recorded)	4930 (99.2)	57,057 (99.4)		2317 (99.2)	2613 (99.1)	
Asthma ^b^						
Yes	435 (8.8)	3627 (6.3)	<0.001	205 (8.8)	230 (8.7)	0.95
No (or not recorded)	4536 (91.2)	53,783 (93.7)		2130 (91.2)	2406 (91.3)	
APLS ^b^						
Yes	11 (0.2)	3 (0.0)	<0.001	5 (0.2)	6 (0.2)	0.92
No (or not recorded)	4960 (99.8)	57,407 (100)		2330 (99.8)	2630 (99.8)	
Thrombophilia ^b,c^						
Yes	60 (1.2)	47 (0.1)	<0.001	39 (1.7)	21 (0.8)	<0.05
No (or not recorded)	4911 (98.8)	57,363 (99.9)		2296 (98.3)	2615 (99.2)	
Heart disease						
Yes	1 (0.0)	7 (0.0)	0.636	0 (0.0)	1 (0.0)	0.35
No (or not recorded)	2970 (100)	57,403 (100)		2335 (100)	2635 (100)	
Kidney disease						
Yes	34 (0.7)	288 (0.5)	0.085	18 (0.8)	16 (0.6)	0.48
No (or not recorded)	4937 (99.3)	57,122 (99.5)		2317 (99.2)	2620 (99.4)	

RPL: recurrent pregnancy loss; BMI: body mass index; SLE: systemic lupus erythematosus; IBD: inflammatory bowel disease; APLS: antiphospholipid syndrome. * Number of pregnancies by control women. Characteristics at each pregnancy in the control group. ^a^ First childbirth after diagnosis with RPL in the National Patient Register; ^b^ *p* < 0.05 = significant difference between women diagnosed with RPL and controls characteristics; ^c^ *p* < 0.05 = significant difference between primary and secondary RPL characteristics.

**Table 2:** (**a**) Obstetrical outcome in the first subsequent pregnancy/childbirth after the diagnosis of recurrent pregnancy loss (RPL) and pregnancies by women without RPL identified in the Swedish Medical Birth Register and the Swedish National Patient Register; (**b**) Obstetrical outcome, in the first subsequent pregnancy after the diagnosis of recurrent pregnancy loss (RPL) and pregnancies by women without RPL, with and without preterm birth.

Obstetrical Outcome	RPL *n* = 4971 (%)	No RPL *n* = 57,410 ** (%)	Crude OR (95% CI)	Adjusted OR (95% CI)
(**a**)				
**Pre-eclampsia** ^a^				
No PE	4757 (95.7)	56,135 (97,8)	1.00 *	1.00 *
Any PE ^1^	214 (4.3)	1275 (2.2)	1.97 (1.70–2.28)	1.45 (1.24–1.69)
No PE			1.00 *	1.00 *
Mild/moderate PE	148 (3.0)	929 (1.6)	1.87 (1.57–2.23)	1.39 (1.17–1.68)
Severe PE ^1^	66 (1.3)	346 (0.6)	2.22 (1.70–2.90)	1.57 (1.20–2.07)
**Stillbirth** ^b^				
Live birth	4939 (99.4)	57,178 (99.6)	1.00 *	1.00 *
Stillbirth	32 (0.6)	232 (0.4)	1.60 (1.10–2.31)	1.38 (0.95–2.02)
**SGA** ^c^				
AGA or LGA	4800 (96.6)	56,001 (97.5)	1.00 *	1.00 *
SGA	171 (3.4)	1409 (2.5)	1.42 (1.21–1.66)	1.25 (1.06–1.47)
**Preterm birth** ^d^				
Term birth ^2^	4475 (90)	54,528 (95)	1.00 *	1.00 *
Preterm birth < 37 GWs ^2^	496 (10)	2882 (5.0)	2.10 (1.90–2.32)	1.95 (1.76–2.16)
Preterm birth 32–36 GWs ^2^	346 (7.0)	2482 (4.3)	1.70 (1.51–1.91)	1.62 (1.43–1.82)
Preterm birth < 32 GWs ^2^	150 (3.0)	398 (0.7)	4.59 (3.80–5.56)	3.86 (3.16–4.72)
Term birth ^2^	4475 (90)	54,528 (95)	1.00 *	1.00 *
Spontaneous preterm birth	281 (5.7)	1886 (3.3)	1.77 (1.56–2.02)	1.82 (1.59–2.08)
Iatrogenic preterm birth	215 (4.3)	996 (1.7)	2.23 (1.73–2.86)	2.16 (1.85–2.53)
Term birth ^2^	4475	54,528	1.00 *	1.00 *
Preterm birth 32–36 GWs ^2^	346 (7.0)	2482 (4.3)	1.70 (1.51–1.91)	1.62 (1.43–1.82)
Preterm birth <32 GWs ^2^	150 (3.0)	398 (0.7)	4.59 (3.80–5.56)	3.86 (3.16–4.72)
**Placental abruption** ^e^				
No placental abruption	4921 (99)	57,200 (99.6)	1.00 *	1.00 *
Placental abruption	50 (1.0)	210 (0.4)	2.77 (2.03–3.77)	2.45 (1.78–3.38)
(**b**)				
**Pre-eclampsia** ^a^				
No PE ***	4759 (95.7)	56,140 (97.8)	1.00 *	1.00 *
PE and preterm	65 (1.3)	248 (0.4)	3.09 (2.35–4.07)	2.26 (1.70–3.01)
PE at term	147 (3.0)	1022 (1.8)	1.70 (1.42–2.02)	1.26 (1.05–1.51)
**Stillbirth** ^b^				
Live birth ***	4939 (99.4)	57,178 (99.6)	1.00 *	1.00 *
Stillbirth and preterm	24 (0.5)	126 (0.2)	2.21 (1.42–3.42)	1.92 (1.22–3.02)
Stillbirth at term	8 (0.2)	106 (0.2)	0.87 (0.43–1.79)	0.76 (0.37–1.57)
**SGA** ^c^				
No SGA ***	4800 (96.6)	56,001 (97.5)	1.00 *	1.00 *
SGA and preterm	75 (1.5)	343 (0.6)	2.55 (1.98–3.28)	2.00 (1.54–2.60)
SGA at term	96 (1.9)	1066 (1.9)	1.05 (0.85–1.30)	0.97 (0.78–1.20)
**Placental abruption** ^d^				
No PA ***	4921 (99)	57,200 (99.6)	1.00 *	1.00 *
PA and preterm	29 (0.6)	123 (0.2)	2.74 (1.83–4.11)	2.47 (1.62–3.76)
PA at term	21 (0.4)	87 (0.2)	2.81 (1.74–4.52)	2.42 (1.49–1.96)

^1^ Including eclampsia; ^2^ any delivery start (spontaneous, induction, Caesarean section). * reference; ** number of pregnancies by control women, *** both birth at term and preterm. PE: pre-eclampsia; SGA: small for gestational age; AGA: appropriate for gestational age; LGA: large for gestational age; GWs: gestational weeks. ^a^ Adjusted for age, parity, assisted conception, body mass index, mother’s country of birth, chronic hypertension, pregestational diabetes, smoking; ^b^ adjusted for age, parity, body mass index, smoking; ^c^ adjusted for age, parity, body mass index, smoking, mother’s country of birth, chronic hypertension; ^d^ adjusted for age, parity, body mass index, smoking; ^e^ adjusted for age, parity, body mass index, smoking, chronic hypertension.

**Table 3:** Obstetrical outcome in women with primary and secondary recurrent pregnancy loss (RPL) in a subsequent pregnancy after the diagnoses of RPL, compared with nulliparous and multiparous without RPL.

Obstetrical Outcome	Primary RPL *n* = 2335 (%)	No RPL (Nullipara) *n* = 24,507 (%)	Crude OR (95% CI)	Adjusted OR (95% CI)	Secondary RPL *n* = 2636 (%)	No RPL (Multipara) *n* = 32,903 (%)	Crude OR (95% CI)	Adjusted OR (95% CI)
**Pre-eclampsia** ^a^								
No PE	2192 (93.9)	23,710 (96.7)	1.00 *	1.00 *	2567 (97.4)	32,430 (98.6)	1.00 *	1.00 *
Any PE ^1^	143 (6.1)	797 (3.3)	1.94 (1.62–2.33)	1.45 (1.20–1.75)	69 (2.6)	473 (1.4)	1.84 (1.43–2.38)	1.42 (1.10–1.85)
No PE			1.00 *	1.00 *			1.00 *	1.00 *
mild/moderate	103 (4.4)	577 (2.4)	1.95 (1.58–2.41)	1.48 (1.19–1.85)	43 (1.6)	347 (1.0)	1.55 (1.12–2.13)	1.23 (0.89–1.70)
Severe ^1^	40 (1.7)	220 (0.9)	1.94 (1.38–2.72)	1.39 (0.98–1.98)	26 (1.0)	126 (0.4)	2.59 (1.69–3.96)	1.89 (1.22–2.92)
**Stillbirth** ^b^								
Live birth	2321 (99.4)	24,405 (99.6)	1.00*	1.00*	2618 (99.3)	32,773 (99.6)	1.00 *	1.00 *
Stillbirth	14 (0.6)	102 (0.4)	1.44 (0.82–2.53)	1.36 (0.76–1.41)	18 (0.7)	130 (0.4)	1.73 (1.06–2.84)	1.45 (0.87–2.40)
**SGA** ^c^								
No SGA	2233 (95.6)	23,649 (96.5)	1.00 *	1.00 *	2567 (97.4)	32,352 (98.3)	1.00 *	1.00 *
SGA	102 (4.4)	858 (3.5)	1.26 (1.02–1.55)	1.15 (0.92–1.43)	69 (2.6)	551 (1.7)	1.58 (1.23–2.03)	1.44 (1.11–1.88)
**Preterm delivery** ^d^								
Term birth	2066 (88.5)	23,005 (93.9)	1.00 *	1.00 *	2409 (91.4)	31,524 (95.8)	1.00 *	1.00 *
Preterm birth < 37 GW	269 (11.5)	1502 (6.1)	1.99 (1.74–2.29)	1.85 (1.60–2.13)	227 (11.5)	1380 (4.2)	2.15 (1.86–2.49)	2.07 (1.78–2.40)
Preterm birth 32–36 GW	170 (7.3)	1290 (5.2)	1.47 (1.24–1.73)	1.41 (1.19–1.68)	176 (6.7)	1192 (3.6)	1.93 (1.64–2.28)	1.86 (1.57–2.20)
Preterm birth < 32 GW	99 (4.2)	211 (0.9)	5.23 (4.10–6.66)	4.14 (3.20–5.37)	51 (1.9)	187 (0.6)	3.57 (2.61–4.88)	3.37 (2.44–4.66)
**Placental abruption** ^e^								
No placental abruption	2309 (98.9)	24,420 (99.6)	1.00 *	1.00 *	2612 (99.1)	32,780 (99.6)	1.00 *	1.00 *
Placental abruption	26 (1.1)	87 (0.4)	3.16 (2.04–4.91)	2.48 (1.57–3.92)	24 (0.9)	123 (0.4)	2.45 (1.68–3.80)	2.42 (1.54–3.79)

^1^ Including eclampsia. * reference. PE: pre-eclampsia; SGA: small for gestational age defined as infant <2 SD; AGA: appropriate for age; LGA: large for age. ^a^ Adjusted for age, smoking, body mass index, assisted conception, mother’s country of birth, chronic hypertension, pre-gestational diabetes; ^b^ adjusted for age, smoking, body mass index; ^c^ adjusted for age, smoking, body mass index, mother’s country of birth, assisted conception; ^d^ adjusted for age, smoking, body mass index, mother’s country of birth, assisted conception, ^e^ adjusted for age, smoking, body mass index.

## Data Availability

Data available on request.
